# Genetic variability within and among populations of an invasive, exotic orchid

**DOI:** 10.1093/aobpla/plv077

**Published:** 2015-07-10

**Authors:** Sueme Ueno, Jucelene Fernandes Rodrigues, Alessandro Alves-Pereira, Emerson Ricardo Pansarin, Elizabeth Ann Veasey

**Affiliations:** 1 Departamento de Genética, Escola Superior de Agricultura ‘Luiz de Queiroz’, Universidade de São Paulo, CP 83, Piracicaba, São Paulo 13418-900, Brazil; 2 Departamento de Biologia, FFCLRP, Universidade de São Paulo, Ribeirão Preto, São Paulo 14040-901, Brazil

**Keywords:** Genetic diversity, invasive plants, ISSR markers, *Oeceoclades maculata*, Orchidaceae, population genetic structure

## Abstract

Despite the fact that invasive species are of great evolutionary interest because of their success in colonizing and spreading into new areas, the factors underlying this success often remain obscure. In this sense, studies on population genetics and phylogenetic relationships of invasive species could offer insights into mechanisms of invasions. Originally from Africa, the terrestrial orchid *Oeceoclades maculata*, considered an invasive plant, is the only species of the genus throughout the Americas. Considering the lack of information on population genetics of this species, the aim of this study was to evaluate the genetic diversity and structure of Brazilian populations of *O. maculata*. We used 13 inter-simple sequence repeat primers to assess the genetic diversity of 152 individuals of *O. maculata* distributed in five sampled sites from three Brazilian states (São Paulo, Mato Grosso and Paraná). Low diversity was found within samples, with estimates of the Shannon index (*H*) ranging from 0.0094 to 0.1054 and estimates of Nei's gene diversity (*H*_e_) ranging from 0.0054 to 0.0668. However, when evaluated together, the sampling locations showed substantially higher diversity estimates (*H* = 0.3869, *H*_e_ = 0.2556), and most of the genetic diversity was found among populations (Φ_ST_ = 0.933). Both clustering and principal coordinate analysis indicate the existence of five distinct groups, corresponding to the sampled localities, and which were also recovered in the Bayesian analysis. A substructure was observed in one of the localities, suggesting a lack of gene flow even between very small distances. The patterns of genetic structure found in this study may be understood considering the interaction of several probable reproductive strategies with its history of colonization involving possible genetic drift, selective pressures and multiple introductions.

## Abstract (precis)

Originally from Africa, the terrestrial orchid *Oeceoclades maculata*, considered an invasive plant, is the only species of the genus throughout the Americas. We used 13 ISSR primers to assess the genetic diversity and structure of 152 individuals of *O. maculata* distributed in five sampled sites in Brazil. Low diversity was found within samples while most of the genetic diversity was found among localities. A substructure was observed in one of the sites, suggesting lack of gene flow even between very small distances. Results may be understood considering the interaction between the reproductive system, colonization history, genetic drift, selective pressures and multiple introductions.

## Introduction

Biological invasions represent one of the major threats to biodiversity, ecosystem integrity, agriculture, fisheries and public health (Lee 2002). Despite the fact that rapid spread of exotics has received considerable attention within the international community, and has mobilized substantial ecological research, the drivers of successful invasion remain poorly understood (Rollins *et al.* 2013). Consequently, incorporating evolutionary genetics is important for revealing characteristics that determine invasion success (Lee 2002).

Although many species of Orchidaceae are endangered due to the destruction and fragmentation of their habitats and the predatory extraction of specimens in natural environments (Muñoz *et al.* 2010), part of the orchids are not at risk of extinction and some, indeed, behave like weeds (Ackerman 2007; Cohen and Ackerman 2009; Liu and Pemberton 2010; De Long *et al*. 2013; García-González *et al.* 2013; Kolanowska 2013, 2014; Ackerman *et al.* 2014; Kolanowska and Konowalik 2014). There is still little information concerning the genetic structure of populations in species of Orchidaceae which behave as colonizers, that is, those that are established in habitats not previously occupied by them (Sun 1997). In addition, there is no consensus on whether it is recommended to tolerate or prevent the establishment of these colonizing orchid species (Ackerman 2007). While it is unlikely that the establishment of invasive orchids cause severe impacts to the new habitat, Recart *et al.* (2013) suggest that even apparently harmless, this process may have a negative impact on native species.

Genetic and evolutionary processes are the main determinants of the establishment and spreading ability of invasive species (Sakai *et al.* 2001). Factors affecting the potential of plants to colonize rapidly and efficiently new habitats include broad environmental tolerance, phenotypic plasticity, inbreeding capacity or any form of asexual reproduction, effective dispersal ability, high relative growth rate and high competitive ability (te Beest *et al.* 2012). Although colonizing new areas usually results in a founder effect and subsequent genetic drift, which reduces the variation in the population and increases the differentiation between them, high levels of diversity are found in many weed species (Ma *et al.* 2011). Introduced species usually exhibit changes in genetic variation, population structure and phenotypic characteristics due to selective pressures that do not occur in conditions of demographic equilibrium, which may reflect previous evolutionary histories, stochastic events and selection (Keller and Taylor 2008). Thus, biological invasions provide valuable opportunities for evolutionary studies that occur in a short period of time (Barrett *et al.* 2008).

The Orchidaceae represents ∼7 % of angiosperms, and is considered one of the largest families of this group (Pridgeon *et al.* 1999). It is characterized for being cosmopolitan, although tropical regions present a higher number of species (Dressler 1981). Although most orchids possess an epiphytic habit, there are also terrestrial, marshy, rupicolous and micro-heterotrophic species (Dressler 1993). Additionally, they present a large diversity of vegetative and floral morphology, exhibiting a great variety of pollination mechanisms, allowing the colonization of different types of environments (Hoehne 1949).

The combination of high dispersal ability, conferred by the morphology of their seeds (species with wind-dispersed seeds), and their preference for ephemeral environments provide a degree of resilience to orchids that is often underestimated (Ackerman 2007). Evaluations of the functions of mycorrhizal fungi and soil factors related to colonization capacity of *Microtis media* R.Br, an invasive terrestrial orchid, support the idea that terrestrial invasive orchids can be associated with the occurrence of a great diversity of mycorrhizal fungi, increasing the chance of seed germination and tolerance to a wide range of habitats, providing, therefore, competitive advantage over native species of orchids (De Long *et al.* 2013). However, it is very difficult to define clear colonization patterns of invasive orchids because they occupy a variety of habitats, have different modes of reproduction and symbiotic relationships (Ackerman 2007; Cohen and Ackerman 2009; De Long *et al*. 2013; Recart *et al.* 2013; Ackerman *et al.* 2014).

*Oeceoclades maculata* was originated from the tropical regions of Africa, as all the other 38 species of the genus, but it is the only species in the genus that occurs in the Neotropics as well (Govaerts *et al.* 2012). The way it was introduced in the Americas remains unknown, it is possible that it was transported to South America through slave trade ships around 1500 (Kolanowska 2013, 2014). Nowadays, *O. maculata* occurs throughout the Neotropics, making it one of the most successful invasive plant species (Stern 1988; Cohen and Ackerman 2009). In Cuba, at the Reserva de la Biosfera Sierra del Rosario, in a wide survey of orchid species, *O. maculata* was the most abundant species (García-González and Márquez 2011). As an old invader, it appears that the African Spotted Orchid, as it is also known, already reached its distribution limits in the Neotropics (Kolanowska 2014). According to this author, climate changes will probably result in shifts in the distribution of the species suitable niches, causing its expansion to new areas, including North America.

The species is characterized by ovoid pseudobulbs with an elliptical apical leaf, and very short rhizomes. It presents erect racemes and its flowers are odourless with sepals and petals of a pink-greenish colour (Aguiar *et al.* 2012). *Oeceoclades maculata* has a terrestrial habit and grows well either in dry or in humid environments, generally occurring in disturbed areas (Ackerman 1995). In fact, it is abundant in environments with moderate disturbance levels (Cohen and Ackerman 2009). In Brazil, the species is widespread and occurs in several types of vegetation (E. R. Pansarin, pers. obs.). The species reproduces vegetatively or by seed (Pabst and Dungs 1977). González-Díaz and Ackerman (1988) found that in Puerto Rico the species shows a passive mechanism of self-fertilization facilitated by rain, which collaborate to explain their wide dispersal in the Americas. However, studies by Aguiar *et al.* (2012) point out that, besides the occurrence of self-pollination mediated by rain, in Brazil the species is pollinated by butterflies, enabling cross-pollination, indicating the existence of variation in reproductive biology of the species along its geographic range.

Cytogenetic studies carried out by Felix and Guerra (2000) and Daviña *et al.* (2009) considered that *Oeceoclades maculata* is octaployd, with *x* = 7 corresponding to the basic number of chromosomes of the tribe. According to Sun (1997), polyploidy is a common feature of several successful colonizing species and can collaborate with a broad environmental tolerance.

Inter simple sequence repeat (ISSR) markers show great potential for studies on genetic diversity and structuring of natural populations (Wolfe *et al.* 1998). Developed by Gupta *et al.* (1994) and Zietkiewicz *et al.* (1994), the ISSR technique is simple, fast and has high reproducibility. These markers have also been used in genetic studies of populations and evolution of Orchidaceae species, such as in *Piperia* (George *et al.* 2009), *Cymbidium* (Wang *et al.* 2009; Sharma *et al.* 2013), *Cattleya* (da Cruz *et al.* 2011; Pinheiro *et al.* 2012; Fajardo *et al.* 2014; Rodrigues *et al.* 2015), *Calanthe* (Qian *et al.* 2013), *Vanda* (Manners *et al.* 2013), *Octomeria* (Barbosa *et al.* 2013) and *Dendrobium* (Feng *et al.* 2013).

Considering the lack of information on the genetic diversity and structure of *Oeceoclades maculata*, this is the first study using molecular markers for this species, and since ISSR markers have proved suitable for such approaches, the objectives of this study were: to characterize the genetic diversity and structure of populations of *O. maculata* by using ISSR molecular markers in order to make inferences about the evolutionary events that act on weed orchids.

## Methods

### Sampling procedures

**Figure 1.**
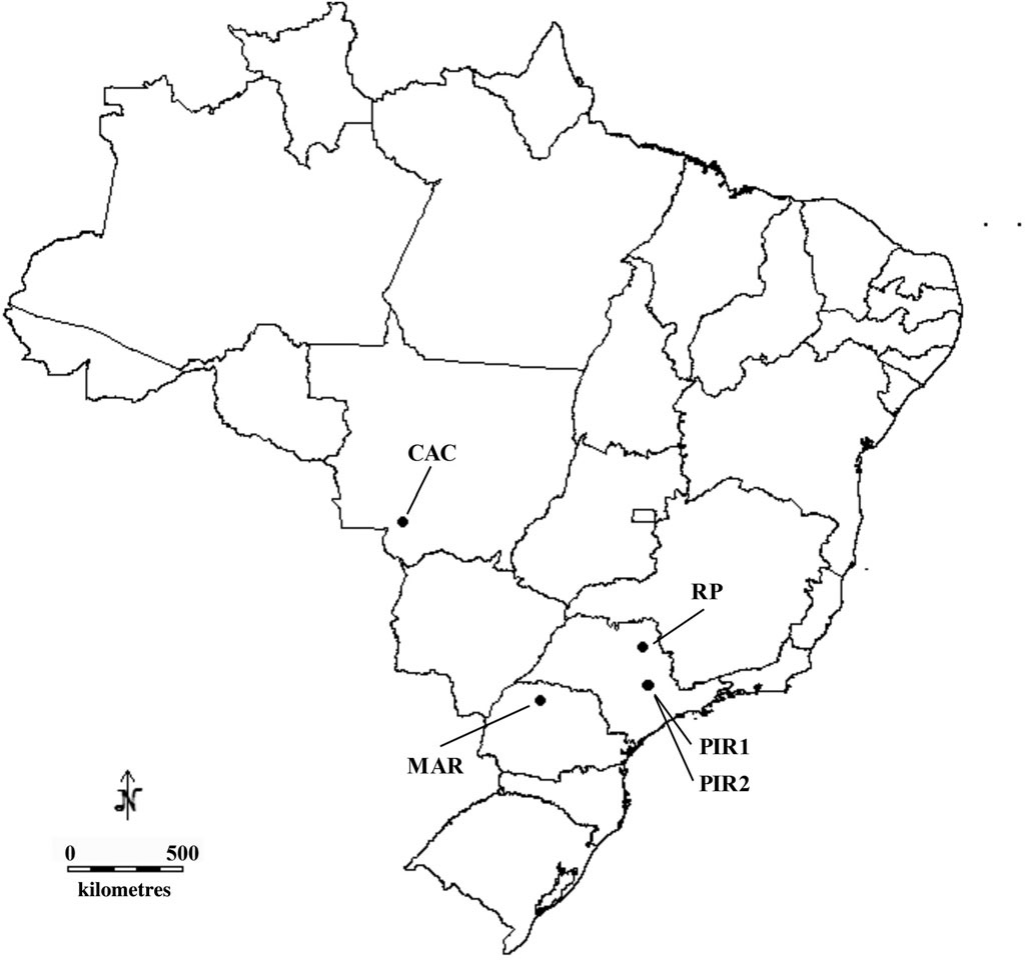
Map of Brazil identifying the sampling sites (points) of *Oeceoclades maculata* collection.

Individual samples were collected in five *Oeceoclades maculata* locations from the Brazilian states of Paraná, in the municipality of Maringá (MAR), Mato Grosso, in the municipality of Cáceres (CAC) and São Paulo, one in the city of Ribeirão Preto (RP) and two in the municipality of Piracicaba (PIR1 and PIR2) (Table 1). In the latter, one sampled location was along the Piracicaba River bank and the other within the campus of Luiz de Queiroz College of Agriculture, University of São Paulo (ESALQ/USP), at a distance of ∼1.25 km between each other. In order to verify if there was any genetic differentiation at a short spatial scale, the sampling within ESALQ/USP was collected at three different locations within the campus, separated by 690 and 480 m. Therefore, we considered these three locations as three subsamples of ESALQ/USP population (Fig. 1). All collection sites were characterized for having their environments disturbed by human activity (close to crop cultivation areas, pasture or urban environments), although in Maringá the vegetation of the sampled site was denser than in the other places. Plant sizes from all sampled sites varied from 12 to 30 cm, showing the same phenotypic characteristics **[see Supporting Information—Fig. S1]**. During the collections geographical coordinates were recorded using the global position system (GPS).

**Table 1.** Origin (municipality-state/collection site), number of sampled individuals (*n*) and geographical coordinates of *Oeceoclades maculata* populations.

Code	Origin	*n*	Coordinates	
MAR	Maringá-PR/Fazenda Experimental Iguatemi	19	23°21′38″S	52°03′53″W
CAC	Cáceres- MT	34	16°02′40″S	57°38′40″W
RP	Ribeirão Preto- SP/Campus of USP	39	21°10′04″S	47°51′19″W
PIR1	Piracicaba1-SP/Rio Piracicaba	21	22°42′11″S	47°38′30″W
PIR2	Piracicaba2-SP/Campus of ESALQ-USP	39	22°42′38″S	47°37′57″W
PIR2.1	Piracicaba-SP/Campus of ESALQ-USP	10	22°42′38″S	47°37′57″W
PIR2.2	Piracicaba-SP/Campus of ESALQ-USP	10	22°42′38″S	47°37′54″W
PIR2.3	Piracicaba-SP/Campus of ESALQ-USP	19	22°42′44″S	47°37′39″W
Total		152

### DNA extraction and SSR protocol

New and healthy leaves were sampled from 19 to 39 individuals per sampled site. The sampled leaves were stored at 1.5 mL microtubes containing CTAB/salt gel (3 % CTAB, 35 % NaCl), and maintained under refrigeration at 4 °C for, at least, 7 days before DNA extraction. The protocol used for DNA extraction was that described by Doyle and Doyle (1987) with minor modifications. DNA was quantified in 1 % agarose gels, run under TBE 10× buffer (90 mM Tris–HCl, 90 mM boric acid and 2 mM EDTA), stained with ethidium bromide. The samples were then purified using Qiagen^®^ QIA PCR purification kit and resuspended in 50 µL of EB buffer (kit Qiagen^®^) following manufacturer's instructions.

A total of 20 ISSR primers (Wolfe 2000) were tested, and 13 primers were selected considering the polymorphism, sharpness and easiness of bands identification: [(CA)7-YC, (GT)7-YG, (AG)7-YC, (CAC)4-RC, (CTC)4-RC, (GAG)4-RC, (GTG)4-RC, (CT)8-TG, (CT)8-RA, (CT)8-RC, (CA)6-RY, (CA)6-RG, (GT)6-AY]. PCR reactions were performed using 10 % (v/v) buffer [20 mM Tris–HCl (pH 8.0), 0.1 mM EDTA, 1 mM DTT, 50 % (v/v) glycerol]; 2.0 or 2.33 mM MgCl_2_, depending on the primer; 0.2 mM dNTPs; 10 pmol of each primer; 1 unit of *Taq* DNA polymerase and 20 ng DNA with a final volume of 30 μL.

The reactions were performed in a MyCycler BioRad thermocycler, according to the following amplification conditions: 94 °C/90 s; 35 cycles of 94 °C/40 s, 44 °C/45 s; 72 °C/90 s; 94 °C/45 s; 44 °C/45 s and a final extension of 72 °C/5 min. The products resulting from the amplification reaction were subjected to electrophoresis on 2 % agarose gel in 1× TBE buffer for 90 min at 135 V. To assist the analysis of the bands stained with ethidium bromide, 3 μg of 100 bp DNA Ladder was used (Life Technologies, USA). The gels were then photographed on a transilluminator with a photo documentation system (Syngene, Synoptics Ltd). The genotyping was performed manually by comparing the banding patterns on the agarose gels. We used only stable, unambiguous, high resolution and reproducible bands.

### Data analysis

Data analysis was performed based on a binary matrix, with presence (1) or absence (0) of bands, considering only the robust and unambiguous bands. Genetic diversity was estimated by the parameters: number of bands, number of polymorphic bands, percentage of polymorphic loci (number of polymorphic bands divided by the total number of bands), number of private bands (which occurred in only one population), number of distinct banding patterns (different band combinations), Nei's genetic diversity (Nei 1978) and Shannon–Wiener diversity index (Shannon 1948). Originally developed for co-dominant data, the diversity index of Nei (1978) is based on the expected heterozygosity (*H*_e_). However, since this concept is not applicable to dominant markers, it becomes a measure of genetic variability estimated by *H*_e_ = 1 − Σ*p_i_*^2^, where *p_i_* is the frequency of a given band in the population. The Shannon–Wiener index (Shannon 1948), which is traditionally used in ecological studies to determine parameters of biodiversity, estimates the degree of certainty of the genetic proximity between individuals. The expression used is *H* =− Σ *p_i_* · log *p_i_*, where *p_i_* is the frequency of a given band in the population. Both genetic diversity parameters (*H*_e_ and *H*) were estimated using POPGENE (Yeh *et al.* 1997).

To evaluate the genetic structure of the populations on the sampled sites, the analysis of molecular variance (AMOVA) (Excoffier *et al.* 1992) was performed using Arlequin v.3.5 (Excoffier and Lischer 2010). Cluster analyses were performed based on Jaccard similarity coefficients and neighbour-joining method using DARwin5 (Perrier *et al.* 2003). The consistency of groupings was verified by 1000 bootstrap replicates. The genetic structure was also evaluated by the principal coordinate analysis (PCoA), using the Euclidean distance with GenAlEx v.6.4 (Peakall and Smouse 2006).

Additionally, in order to investigate how the sampled sites are structured, Bayesian analysis was performed using Structure v.3.3 (Pritchard *et al.* 2000; Falush *et al.* 2007) to infer the groups of populations or individuals that were genetically more related. Ten independent simulations for each number of *K* groups (with *K* ranging from 1 to 10) were performed. Each simulation consisted of 500 000 iterations of MCMC after an initial discard (burn-in) of 250 000. The analysis was performed using the no admixture ancestry model and the no correlated allele frequencies model, since the species is considered to be self-pollinated. The most probable number of groups was estimated by the *ad hoc* method based on Δ*K* (Evanno *et al.* 2005).

To investigate the possibility of isolation by distance among sampled locations, the Mantel test (Mantel 1967) was performed in Arlequin v.3.5 (Excoffier and Lischer 2010). The correlation was estimated between two matrices, one resultant from genetic dissimilarities estimates between all possible pairs of locations and the other resulting from geographic distances between pairs of locations.

## Results

The 13 ISSR primers generated a total of 192 bands, ranging from 6 to 24 bands per primer, of which 189 were polymorphic (considering that the frequency of the most common allele does not exceed 95 %). Considering each sampled site, the number of polymorphic bands ranged from 7 (PIR1 and RP) to 55 (PIR2) (Table 2). Piracicaba2/SP (PIR2) sampled site had the highest number of private bands (23), while CAC had only five. The polymorphism percentage within sampled sites ranged from 3.7 % (PIR1 and RP) to 27.6 % (PIR2), with an average of 10 %, indicating low levels of intra-population variability. However, when considering all sampled locations, a high percentage of polymorphic bands (85.9 %) was found (Table 2). With respect to the number of distinct banding patterns, a total of 81 banding patterns was observed in the sampled sites, ranging from 8 (PIR1) to 34 (PIR2).

**Table 2.** Genetic diversity estimates of *Oeceoclades maculata* populations including number of individuals (*N*), number of bands (≥5 %) (NB), number of polymorphic bands (NPB), percentage of polymorphic bands (*P*), number of private bands (NPRB), number of distinct banding patterns (BP), Shannon index (*H*) and estimates of Nei's gene diversity (*H*_e_). Standard deviations are in parentheses.

Populations	*N*	NB	NPB	*P*	NPRB	BP	*H*	*H*_e_
MAR	19	97	15	9.90	15	11	0.0378 (0.134)	0.0241 (0.091)
CAC	34	85	10	5.21	5	17	0.0120 (0.061)	0.0065 (0.036)
RP	39	85	7	3.65	11	11	0.0094 (0.059)	0.0054 (0.036)
PIR1	21	92	7	3.65	13	8	0.0167 (0.094)	0.0110 (0.064)
PIR2	39	108	55	27.60	23	34	0.1054 (0.205)	0.0668 (0.136)
Mean	30.4	95.4	18.8	10.00	13.4	16.2	0.0362 (0.111)	0.0228 (0.073)
Total	152	192	189	85.94	67	81	0.3869 (0.257)	0.2556 (0.185)

The Shannon index (*H*) estimates were low, ranging from 0.0094 (RP) to 0.1054 (PIR2), as well as the estimates of Nei's genetic diversity, ranging from 0.0054 (RP) to 0.0668 (PIR2). However, when evaluated together, the five sampling sites presented substantially higher estimates for the Shannon index and Nei's genetic diversity (*H* = 0.3869; *H*_e_ = 0.2556) (Table 2). The AMOVA indicated a strong genetic structure among sampled sites (Table 3), with a Φ_ST_ value of 0.933. Only 6.7 % of genetic variation revealed by ISSR markers was due to differences between individuals within sampled sites. The number of distinct banding patterns found within sampled sites (Table 2) confirms the results of AMOVA, Shannon index and Nei's genetic diversity. Results showed that the sampled sites do not share any of the 81 banding patterns found. Moreover, at least one banding pattern was shared by more than one individual within each location.

**Table 3.** Analysis of molecular variance between and among *Oeceoclades maculata* populations. ^1^Degrees of freedom; ^2^Sum of squares; ^3^*P* (1023 permutations) = 0.0000.

Source	DF^1^	SS^2^	Variance components	Total variation (%)^3^
Among populations	4	3425.259	29.316	93.34
Within populations	147	303.303	2.092	6.66
Total	151	3728.562	31.409	
Φ_ST_	0.933			

**Figure 2.**
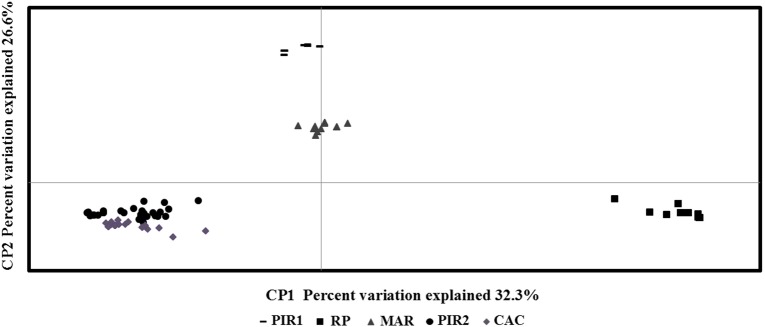
Principal coordinates analysis of 152 individuals of *Oeceoclades maculata*, based on the genetic variation revealed by ISSR markers. Individuals are classified according to the populations of origin: Ribeirão Preto/SP (RP), Cáceres/MT (CAC), Piracicaba1/SP (PIR1), Piracicaba2/SP (PIR2) and Maringá/PR (MAR).

**Figure 3.**
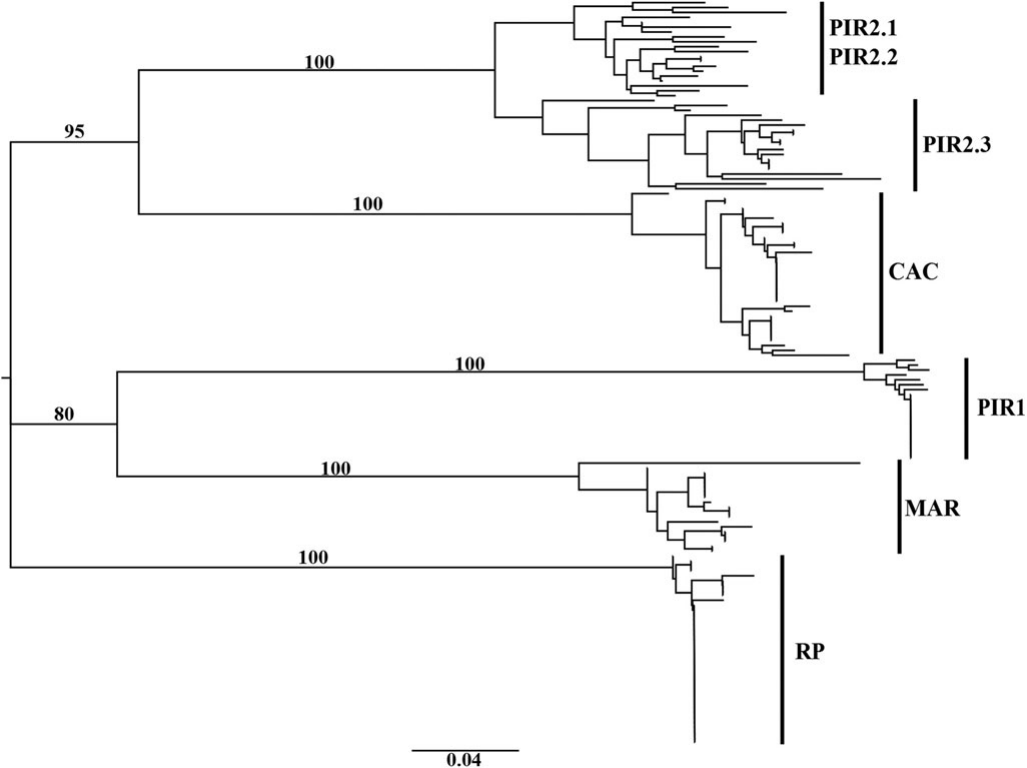
Dendrogram obtained with the neighbour-joining algorithm, based on Jaccard dissimilarity for five populations [Ribeirão Preto/SP (RP), Cáceres/MT (CAC), Piracicaba1/SP (PIR1), Piracicaba2/SP (PIR2.1, PIR2.2, PIR2.3) and Maringá/PR (MAR)] of *Oeceoclades maculata*.

The PCoA showed that 58.2 % of the variation is accumulated in the first two axes, revealing the existence of significant genetic differences among sampled locations (Fig. 2). The RP location was the most isolated, while a greater proximity was observed between PIR2 and CAC locations and between Piracicaba1/SP (PIR1) and MAR sampled sites. This result is consistent with the Mantel test, which showed no significant correlation between genetic and geographic distances (*r*^2^ = 0.0965, *P* = 0.7998). The existence of significant genetic diversity among sampled locations was also observed in the clustering analysis (Fig. 3). The dendrogram is composed of five distinct groups that correspond to the five locations sampled. The branches that separate the groups showed high consistency, with bootstrap values >80 %. Like PCoA, there is greater proximity between PIR2 and CAC locations, and between MAR and PIR1 sampled locations.

The most likely number of clusters estimated with the Bayesian analysis performed with Structure was five **[see Supporting Information—Fig. S2]**. The genetic clusters identified with *K* = 5 were formed according to each sampled location, without the presence of potentially hybrid or migrant individuals. This result corroborates those obtained by PCoA and by clustering analysis. Due to the division of individuals from PIR2 in two groups in the dendrogram, a new Bayesian analysis was performed with only the individuals from this location, with *K* ranging from 1 to 10, and using the same models and number of interactions of MCMC and burn-in to verify the existence of substructuring. The results showed that the more likely number of groups was *K* = 2, confirming the division of the two subgroups in a dendrogram (PIR2.2 and PIR2.3 vs PIR2.1), with the presence of a possible migrant individual **[see Supporting Information—Fig. S3]**.

## Discussion

### Low genetic diversity in populations of *Oeceoclades maculata*

Genetic analysis performed with ISSR markers indicated that *O. maculata* sampled sites from different regions in Brazil have low intra-population diversity. The occurrence of individuals with identical banding patterns certainly contributed to the low genetic diversity indices found within sampled sites. Among the sampled locations evaluated, PIR2 showed greater genetic diversity and the highest number of distinct banding patterns. On the other hand, RP showed the lowest genetic diversity and lowest number of distinct banding patterns. The lower diversity of RP, with a higher sample size, may be due to its presence in a more disturbed area, when compared with, for example, Maringá, with a lower number of individuals sampled, but situated in a neighbouring area of a less disturbed forest.

The low genetic diversity found within *O. maculata* sampled locations corroborated with what is expected from a predominantly autogamous invasive species or of vegetative reproduction, in which uniparental reproduction allows the establishment and spread of extremely small founding groups (Barrett *et al.* 2008), that presumably have lower levels of genetic diversity than the source population. Furthermore, in orchids the low intra-population genetic diversity can be the result of founding events, which lead to small effective population size (*N*_e_), and, therefore, favour the occurrence of genetic drift, in addition to selection pressures that favour a few lineages in a particular environment (Richards 1990; Tremblay *et al*. 2005).

In spite of evidences of variation in the species reproductive biology and the possibility of cross-pollination in Brazilian populations of *O. maculata* by butterflies (Aguiar *et al.* 2012), the genetic diversity levels suggest that self-fertilization, as pointed out by González-Díaz and Ackerman (1988), and vegetative propagation are the predominant forms of reproduction in the studied populations. The low intra-population diversity indicates that even individuals that may have originated from outcrossing are very similar to each other due to the occurrence of inbreeding. However, studies employing co-dominant markers are needed to distinguish the relative influence of these possible explanations in the reproductive dynamics of the populations.

The low intra-population diversity is expected in predominantly autogamous species, as in *O. maculata*, since self-fertilization decreases the proportion of heterozygous loci in individuals, leading to fixation of homozygous loci (Hamilton 2009). Furthermore, the potential for vegetative reproduction through pseudobulbs (Pabst and Dungs 1977) can lead to the maintenance of low genetic diversity levels within populations. Several studies reported low intra-population diversity as a consequence of inbreeding and vegetative reproduction (Mable and Adam 2007; Brütting *et al.* 2012; Sletvold *et al.* 2012; Yang *et al.* 2012; Chen *et al.* 2014). However, not all self-fertilized plants necessarily show low intra-population diversity. In the case of the predominantly inbreeding weed, characterized by greatest colonizing ability, *Capsella bursa-pastoris* (Neuffer *et al.* 2011), genetic differentiation was found both between individuals and among accessions from different populations, due to life-history traits (flowering time, fecundity and dormancy), but also to genetic drift effects and selection to varying environmental conditions (different cropping practices and soil-pH) (Begg *et al.* 2012).

The form of colonization of *O. maculata* (Cohen and Ackerman 2009; Kolanowska 2013) may also have influenced the diversity levels found in this study. Because biological invasions involve dispersal of genes over long distances, the genetic composition of an introduced species is generally influenced by the history of introduction, genetic drift, population size, reproductive system and environmental heterogeneity (Ye *et al.* 2004; Begg *et al.* 2012). Gaskin *et al.* (2012) observed minimum levels of genetic diversity in pepper grass weed populations (*Lepidium latifolium*) and suggested that they were due to founder effects or strong genetic bottlenecks before or after the dispersal events. Sun and Wong (2001), using RAPD markers, reported low Shannon diversity indices (0–0.054) in orchid populations of *Zeuxine strateumatica.* Reproduction through apomixis added to the fact that it is a colonizer species may have led to decreased recombination and gene flow and quick apomictic genotypes fixation (Sun and Wong 2001).

The positive correlation between performance and intra-population genetic variation is valid for outcrossing plants, but these variables are independent for autogamous species because they do not suffer the deleterious effects of inbreeding (Leimu *et al.* 2006). In addition, the genetic diversity of weeds may not be a good predictor of success in colonization (Sakai *et al.* 2001). Field observations and information on the rapid spread of *O. maculata* in tropical and subtropical regions of the Americas (Stern 1988; Aguiar *et al.* 2012), to the point of being considered by many as a Brazilian native species (Morrison 1997; Aguiar *et al.* 2012), suggest that it is a successful colonizer species.

### High genetic structure among *Oeceoclades maculata* populations

The genetic structure analysis of *O. maculata* showed high genetic differentiation among the sampled locations. Analysis of molecular variance results indicate that most of the genetic variation (Φ_ST_ = 0.933) was found between locations, consistent with the low intra-population analyses suggesting predominant self-fertilization. In orchids, the common restriction to gene flow among populations, the usually reduced effective population size and different selection pressures among populations could lead to a high genetic population substructure (Tremblay *et al.* 2005). Although orchids may present high dispersal ability, conferred by the morphology of their seeds (species with wind-dispersed seeds; Ackerman 2007), in plants, seeds dispersion contributes to both the colonization of new populations and for connectivity established between populations, while pollen contributes to the connectivity. Despite the fact that diploid seeds may disperse two times more genes than haploid pollen, pollen movement contributes more to gene flow in most species (Petit *et al.* 2005; Kartzinel *et al*. 2013). Therefore, the results suggest that seed dispersal of *O. maculata* is more efficient than pollen dispersal due to colonization events of new areas.

According to the estimates of Nybom and Bartish (2000), the average value of Φ_ST_ for autogamous species using molecular markers is 0.70. Since estimates of Φ_ST_ obtained by RAPD can be directly compared with those obtained by ISSR (Nybom 2004), the differentiation index found for the populations of *O. maculata* are of similar magnitude to the average value for autogamous populations. Comparing the Φ_ST_ value of 0.86 found in populations of *Stipa capillata* (Poaceae) with the estimated mean value for autogamous species, Hensen *et al.* (2010) suggest that the species has experienced strong genetic bottlenecks, reproductive isolation and self-fertilization. Forrest *et al.* (2004) explained the strong differentiation found among European populations of *Spiranthes romanzoffiana* (Orchidaceae) (Φ_ST_ = 0.892) as resulting from the lack of gene flow between the evaluated populations, combined with different reproductive strategies across populations (autogamy, vegetative reproduction and crossing). Other works with autogamous species (Nybom and Bartish 2000; Xiao *et al.* 2006; Zhang *et al.* 2006; Voss *et al.* 2012; Aguayo *et al.* 2013) found similar values of genetic differentiation to the estimated mean value.

However, unlike the results observed for *O. maculata*, Phillips *et al.* (2012) found that in comparison with other plant families, orchid species present lower mean values of genetic differentiation between populations (*F*_ST_ = 0.146, average for allozymes). The highest values of genetic structure are observed for rare species of terrestrial orchids, but these values are still lower than those of other families. This information, however, contradicts the theory that genetic drift plays an important role in diversification of this family (Tremblay *et al.* 2005). But the fact that there is little genetic diversity within populations of *O. maculata* implies on low effective population size, suggesting that genetic drift may have exerted great influence on the divergence between populations of this species (Slatkin 1987), although more studies are needed to corroborate this hypothesis.

High number of private alleles was found in almost all studied locations, suggesting lack of gene flow and the action of genetic drift. However, the genetic drift inference in our study should be taken with care because the number of sampled sites could not be sufficiently representative for these considerations. The presence of private alleles is important because it may indicate different evolutionary trajectories (Senior *et al.* 1998; Yang *et al.* 2013). Based on genetic diversity data from native populations of *Alliaria petiolata* (Brassicaceae), combined with the genetic diversity indices associated to total allelic richness of colonizing populations, which is intimately related to the amount of private alleles, Durka *et al.* (2005) reported that the colonizing populations would be the result of multiple introductions from several native populations. Unfortunately, there is no information available on the allelic richness of native populations of *O. maculata* that allows similar inferences. However, the presence of genetic structure among sampled sites suggests the occurrence of multiple introductions. Sun (1997) suggests that the low levels of genetic diversity observed among populations of three species of colonizing orchids (*Eulophia sinensis*, *Spiranthes hongkongesis* and *Zeuxine strateumatica*) may be a consequence of the colonization by few individuals or by related individuals. However, contrary to this result, the relevant genetic divergence found between *O. maculata* sampled sites may suggest the colonization by unrelated individuals.

Different selection pressures or stochastic events may lead to remarkable differences among populations that reproduce predominantly by self-pollination. In invasive autogamous populations these differences can occur even over short distances (Richards 1990). The PCoA and the dendrogram showed that the five locations evaluated formed distinct groups without the presence of individuals from other sampled localities. Both analyses showed greater genetic similarity between the sampled sites of PIR2 and CAC and between those of MAR and PIR1, while the RP location remained genetically distinct in both analyzes. Besides, the dendrogram suggested a substructure within the PIR2 sampled site. This result suggests lack of gene flow even between very small distances, contributing to substructuring within *O. maculata* populations. Genetic drift was proposed as a possible cause for the differentiation among genetically isolated but locally co-occurring accessions of the weed *Capsella bursa-pastoris*, promoted by bottlenecks associated with founder events or extinction-colonization dynamics at a field scale (Begg *et al.* 2012). Although further studies would be advisable, genetic drift could be a possible cause for this high genetic differentiation found in *O. maculata* from such small distance sub-populations.

Bayesian analysis corroborated the principal coordinate and clustering analyses, in which the most likely number of groups (*K* = 5) shows that individuals are divided into populations according to the sampled sites, without the presence of putative hybrids or migrant individuals across populations. However, due to substructure observed in the PIR2 population, a new analysis with Structure software showed the division of this population into two subpopulations, with the presence of a potential migrant individual. The potential migrant indicates the existence of certain continuity within this population, and that individuals may be not completely isolated, as occurs when all the five populations are considered. This population also presented the highest intra-population diversity and the dendrogram suggests that even the subpopulations exhibit some variability. This variability suggests differential strategies related to reproduction mode within this population, or even multiple sources of introduction.

### Absence of correlation between genetic and geographic distances

Knowledge on the spatial distribution of genetic diversity is important for a better understanding of the relationships between life-history characteristics, stochastic factors, gene flow, selection pressures and environmental influences (Escudero *et al.* 2003). There was no evidence of the occurrence of isolation by distance among the locations sampled of *O. maculata*, although we should look at these results with caution due to our limited sample size (*N* = 5), a bit too low to infer the relationship between geographic and genetic distances. However, the absence of correlation corroborates the results obtained by the principal coordinates and grouping analysis, which indicated greater genetic similarity between spatially distant populations. Guggisberg *et al.* (2012) suggested that the lack of correlation between genetic and geographic distances of invasive populations of *Cirsium arvense* (Asteraceae) is due to multiple events of introduction and casual dispersal events, mediated by human action. According to the author's observations, *O. maculata* usually occurs in disturbed forests. In preserved areas it does not appear. However, the forest needs to be present. Thus, apparently the dispersion movement is driven by human movements, since it is associated to forest disturbance, but the species does not support the complete forest destruction. Therefore, the absence of correlation between genetic and geographic distances of *O. maculata* populations suggests that the spatial distribution of genetic diversity of this species may be influenced by their reproductive system and history of colonization by seed dispersal, which can be carried through long distances, since they are dispersed by wind (Dressler 1993). This result also reinforces that the *O. maculata* populations may have been originated by different introduction events.

## Conclusions

The genetic diversity and structure analyses using ISSR molecular markers revealed that the studied populations of terrestrial and invasive orchid *O. maculata* have low intra-population genetic diversity and are remarkably divergent among each other. Self-fertilization, drift events, colonization by one or a few individuals, different selection pressures, even at small geographic areas, and multiple introductions may have influenced the genetic diversity and the distribution of the Brazilian *O. maculata* populations. This was a preliminary study on this invasive orchid species, and further studies including a wider sample in different locations of the country would be encouraged. Also, the use of co-dominant markers and sequence data from cpDNA or nrDNA (ITS), would be suitable and would allow us to understand the plant dispersion mechanisms, examining gene flow through seeds and pollen. And also, it would allow us to expand our knowledge on the patterns of population biology of invasive orchids.

## Sources of Funding

We thank the Coordenação de Aperfeiçoamento de Pessoal de Nível Superior (CAPES), Conselho Nacional de Desenvolvimento Científico e Tecnológico (*CNPq*) and Fundação de Amparo à Pesquisa do Estado de São Paulo (FAPESP) for scholarships funding.

## Contributions by the Authors

S.U. designed the study, carried out fieldwork, laboratory work, data analysis and writing; J.F.R. helped in designing the study, fieldwork, laboratory work and writing; A.A.-P. helped in the data analysis and writing; E.R.P. helped in designing the study, fieldwork and writing; E.A.V. helped in designing the study, fieldwork, data analysis and writing.

## Conflict of Interest Statement

None declared.

## Supporting Information

The following additional information is available in the online version of this article —

**Figure S1.** Detail of an *Oeceoclades maculata* plant within a forest fragment at the campus of ESALQ, Piracicaba, SP.

**Figure S2.** Bayesian analysis of the genetic structure of 152 individuals from five populations [Ribeirão Preto/SP (RP), Cáceres/MT (CAC), Piracicaba1/SP (PIR1), Piracicaba2/SP (PIR2) and Maringá/PR (MAR)] of *Oeceoclades maculata* with *K* = 5.

**Figure S3.** Bayesian analysis of the genetic structure of 39 *Oeceoclades maculata* individuals from the ESALQ/USP population, with *K* = 2. Subpopulations are coded following Table 1.

Additional Information
