# Supplementary material for: Genetic variability within and among populations of an invasive, exotic orchid
Source: AoB Plants. 2015 Jul 10;7:plv077. doi: 10.1093/aobpla/plv077 (PMC4564003; doi:10.1093/aobpla/plv077)
Supplement: Additional Information [file supp_7_plv077_index.html]

Genetic variability within and among populations of an invasive, exotic orchid — Additional Information 

# Genetic variability within and among populations of an invasive, exotic orchid

## Additional Information

Additional Information

- Additional Information - Docx file
