# Supplementary material for: Genetic variability within and among populations of an invasive, exotic orchid
Source: AoB Plants. 2015 Jul 10;7:plv077. doi: 10.1093/aobpla/plv077 (PMC4564003; doi:10.1093/aobpla/plv077)
Supplement: Additional Information [file supp_plv077_plv077supp.docx]

**SUPPORTING INFORMATION**


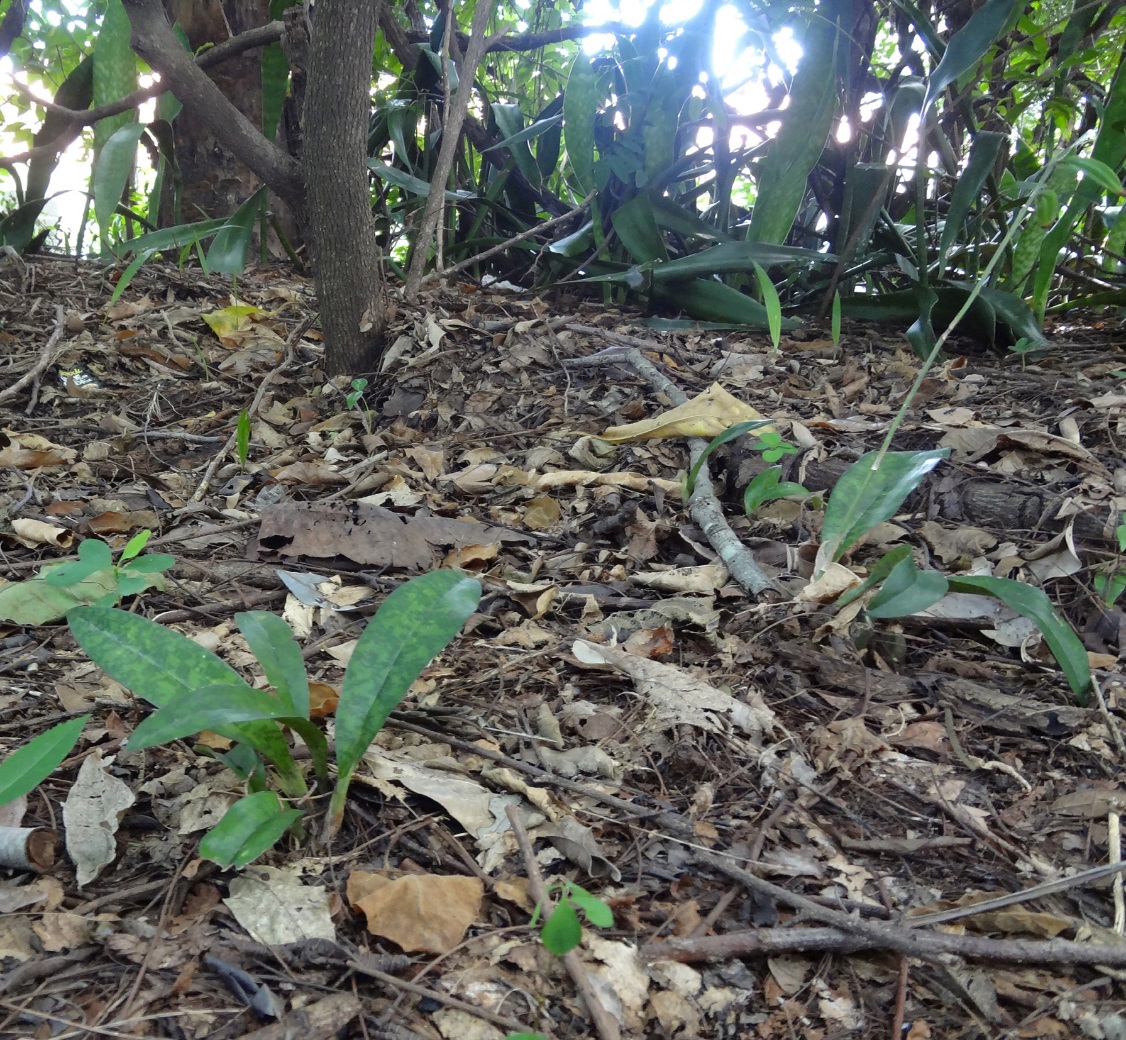


**Figure S1.** Detail of an *Oeceoclades maculata* plant within a forest fragment at the campus of ESALQ, Piracicaba, SP.

**
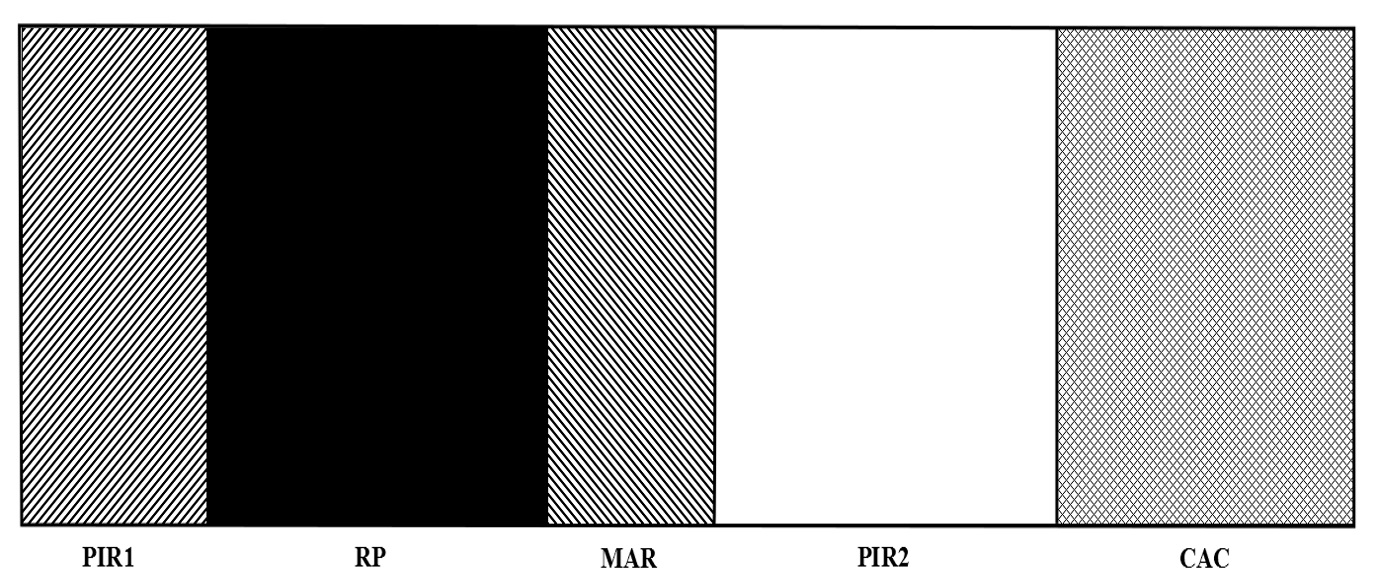
**

**Figure S2.** Bayesian analysis of the genetic structure of 152 individuals from five populations [Ribeirão Preto/SP (RP), Cáceres/MT (CAC), Piracicaba1/SP (PIR1), Piracicaba2/SP (PIR2) and Maringá/PR (MAR)] of *Oeceoclades maculata* with *K* = 5.


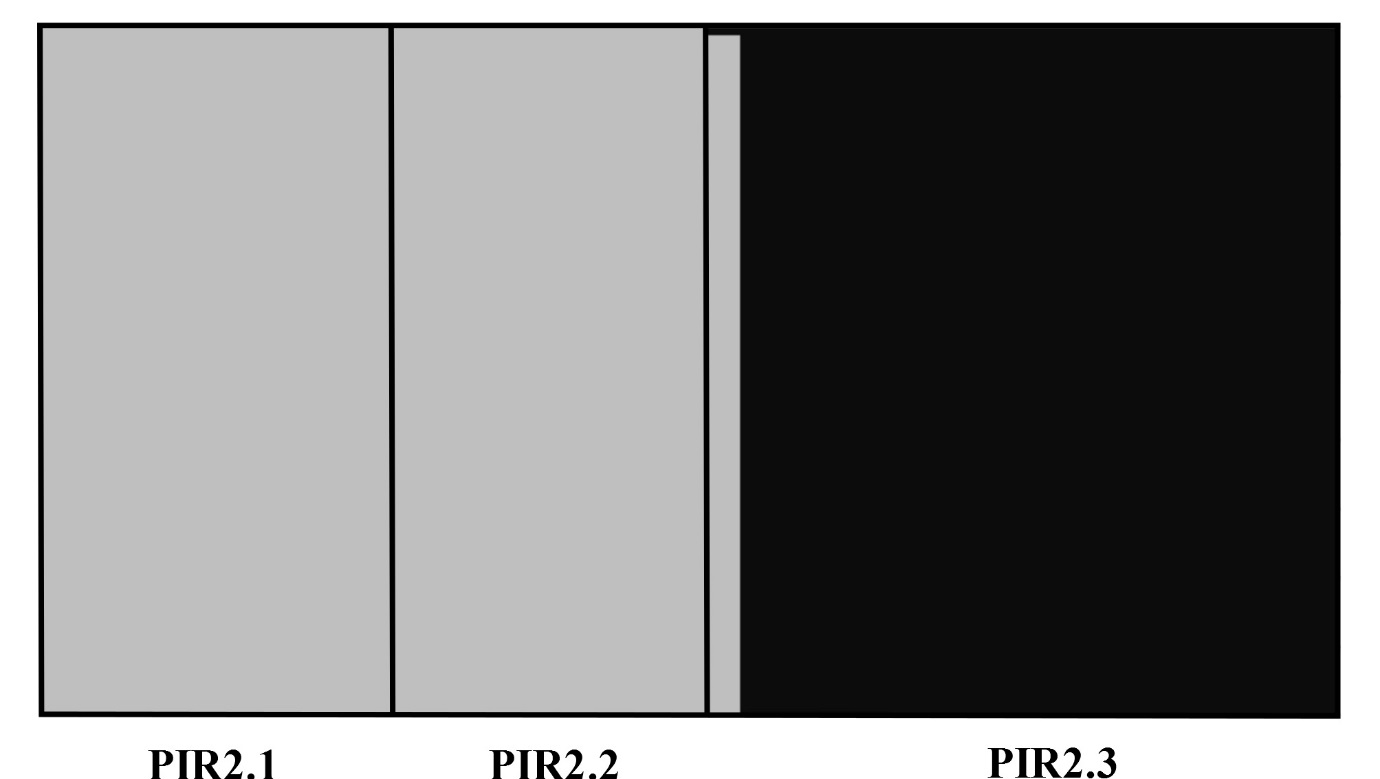


**Figure S3**. Bayesian analysis of the genetic structure of 39 *Oeceoclades maculata* individuals from the ESALQ/USP population, with *K* = 2. Subpopulations are coded following Table 1.
